# Integrative bioinformatics analysis to explore a robust diagnostic signature and landscape of immune cell infiltration in sarcoidosis

**DOI:** 10.3389/fmed.2022.942177

**Published:** 2022-11-04

**Authors:** Mengjie Duo, Zaoqu Liu, Pengfei Li, Yu Wang, Yuyuan Zhang, Siyuan Weng, Youyang Zheng, Mingwei Fan, Ruhao Wu, Hui Xu, Yuqing Ren, Zhe Cheng

**Affiliations:** ^1^Department of Respiratory and Critical Care Medicine, The First Affiliated Hospital of Zhengzhou University, Zhengzhou, China; ^2^Department of Interventional Radiology, The First Affiliated Hospital of Zhengzhou University, Zhengzhou, China; ^3^Interventional Institute of Zhengzhou University, Zhengzhou, China; ^4^Interventional Treatment and Clinical Research Center of Henan Province, Zhengzhou, China; ^5^Department of Cardiovascular Medicine, The First Affiliated Hospital of Zhengzhou University, Zhengzhou, China

**Keywords:** sarcoidosis, diagnostic model, WGCNA, machine learning, immune infiltration, functional analysis, biomarker

## Abstract

**Background:**

The unknown etiology of sarcoidosis with variable clinical features leads to delayed diagnosis and limited therapeutic strategies. Hence, exploring the latent mechanisms and constructing an accessible and reliable diagnostic model of sarcoidosis is vital for innovative therapeutic approaches to improve prognosis.

**Methods:**

This retrospective study analyzed transcriptomes from 11 independent sarcoidosis cohorts, comprising 313 patients and 400 healthy controls. The weighted gene co-expression network analysis (WGCNA) and differentially expressed gene (DEG) analysis were performed to identify molecular biomarkers. Machine learning was employed to fit a diagnostic model. The potential pathogenesis and immune landscape were detected by bioinformatics tools.

**Results:**

A 10-gene signature SARDS consisting of *GBP1, LEF1, IFIT3, LRRN3, IFI44, LHFPL2, RTP4, CD27, EPHX2*, and *CXCL10* was further constructed in the training cohorts by the LASSO algorithm, which performed well in the four independent cohorts with the splendid AUCs ranging from 0.938 to 1.000. The findings were validated in seven independent publicly available gene expression datasets retrieved from whole blood, PBMC, alveolar lavage fluid cells, and lung tissue samples from patients with outstanding AUCs ranging from 0.728 to 0.972. Transcriptional signatures associated with sarcoidosis revealed a potential role of immune response in the development of the disease through bioinformatics analysis.

**Conclusions:**

Our study identified and validated molecular biomarkers for the diagnosis of sarcoidosis and constructed the diagnostic model SARDS to improve the accuracy of early diagnosis of the disease.

## Introduction

Sarcoidosis is a systemic autoimmune disease characterized by non-caseous necrotizing epithelioid granulomas that can affect various organs and tissues such as the lung, eye, skin, heart, and nervous system, with a predominance in young and middle-aged people (1, 2). About 25% of these patients present a chronic, progressive process. Eventually, it can lead to irreversible pathologies, including pulmonary fibrosis, cirrhosis, fatal arrhythmias, and blindness, seriously affecting patients' life quality and longevity (3, 4). Sarcoidosis has a mortality rate of ~7% over a 5-year follow-up period (5). However, patients have significant heterogeneity in the tissues and organs involved, clinical manifestations, responses to treatment, and prognosis, leading to the diagnosis of sarcoidosis relying on a comprehensive assessment of clinical presentation, imaging, and pathology characteristics. Invasive methods such as pathological biopsies are still constrained by samples' accessibility, resulting in delayed diagnosis. Therefore, easily accessible diagnostic approaches are necessary to help patients be diagnosed as early as possible before irreversible pathology to avoid delaying the optimal time for treatment. Given that the etiology of sarcoidosis has not been elucidated, the first line of therapy for sarcoidosis patients is oral glucocorticoids (5), despite their severe side effects (6).

With the rapid advances in bioinformatics, the assessment of blood transcriptional signature may provide a fast, easily accessible, and convenient screening approach to identify potential molecular biomarkers for diagnosing disease and explore the latent pathogenesis and immunological characteristics to provide additional therapeutic perspectives for better individual treatment. Molecular biomarkers from the blood transcriptome are widely used for disease diagnosis and pathogenesis exploration. Several studies have documented that gene expression profiling of peripheral blood could be used as biomarkers in multisystem diseases and immune-related disorders (7–11), like monitoring multiple sclerosis progression (12) and response to treatment and distinguishing active tuberculosis from other infectious and inflammatory diseases (13).

Our study collected 11 microarray cohorts of sarcoidosis patients from the Gene Expression Omnibus (GEO). Our study is committed to identifying specific gene profiles correlated with sarcoidosis through bioinformatics analysis and constructing a robust sarcoidosis diagnostic signature (SARDS). Additionally, the results might provide new insights into the pathogenesis, immune characteristics, and potential treatment options for sarcoidosis.

## Methods

### Data collection and processing

**Figure 1:**
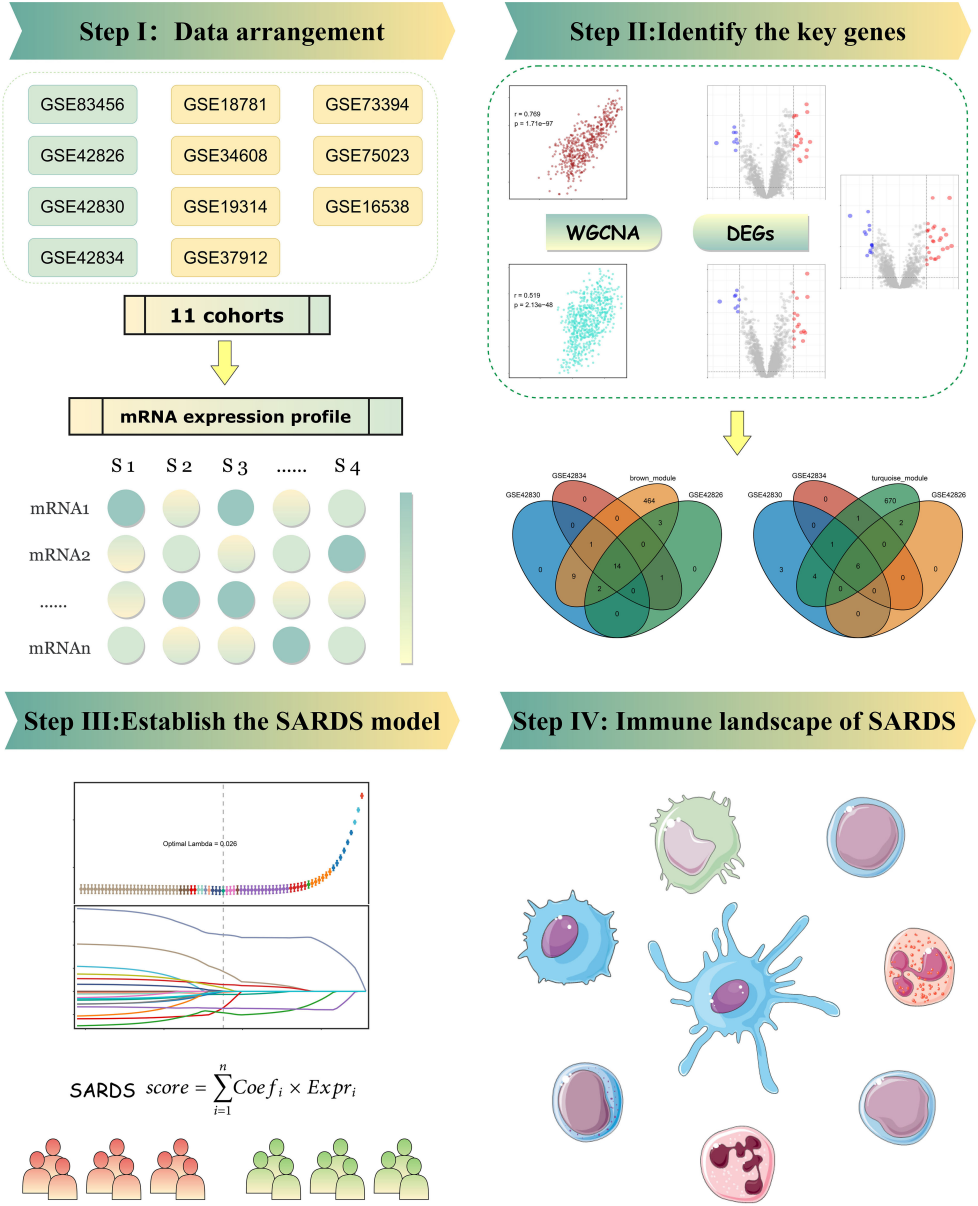
The flowchart of this study.

We downloaded the gene expression profile from GEO (http://www.ncbi.nlm.nih.gov/geo/) by searching “sarcoidosis.” The inclusion criteria were as follows: (i) The datasets contained total RNA gene expression microarray data; (ii) the datasets included sarcoidosis and normal samples: the samples can be one of the five forms, including whole blood, peripheral blood mononuclear cells (PBMC), bronchoalveolar lavage (BAL) cells, and lung tissue; and (iii) the datasets had five samples of both sarcoidosis and normal patients at least. The data processing procedure of the research was illustrated in the workflow (Figure 1).

### Weighted gene co-expression network analysis

The consensus WGCNA approach was employed to cluster genes with similar expression patterns and filter out clusters of co-expressed genes called “modules” which are highly associated with sarcoidosis via the “WGCNA” R package (14). First, the expression of genes was ranked by standard deviation, and the top 5,000 genes were selected for the subsequent analysis. Next, hierarchical cluster analysis was performed to determine whether there were outlier samples. Soft-thresholding power was based on scale-free analysis and set as the lowest power with a scale-free topology model fit >0.9 by the “pickSoftThreshold” function in the “WGCNA” R package. The soft threshold and the gene similarity matrix calculated by Pearson correlation values between each gene pair were used to construct the adjacency matrix. Subsequently, the adjacency matrix was transformed into a topological overlap matrix (TOM) and a 1-TOM, reflecting the similarity and dissimilarity between genes separately. MinModuleSize was set to 50 to ensure each module had a minimum of 50 genes. Deep Split was set to two to identify modules using dynamic tree cut. MEDissThres was set to 0.2 to cluster module eigen genes (MEs). These genes were classified into different MEs calculated to represent the gene expression. We calculated the correlation between diagnosis and MEs to screen key modules for further analysis. The genes with high gene significance and module membership were considered essential genes.

### Functional enrichment analysis

The functional enrichment analysis contained Gene Ontology (GO) enrichment analysis and Kyoto Encyclopedia of Genes and Genomes (KEGG) pathways enrichment analysis. The biological processes, cellular components, molecular functions, and relevant pathways of the genes in the key MEs were implemented via the “clusterProfiler” R package (15). According to the Benjamini–Hochberg procedure, the *P*-adjusted value was further computed. Set *P*-adjusted value < 0.05 as statistically significant.

### Identification of key regulative genes

The differentially expressed genes (DEGs) between sarcoidosis and normal blood samples were screened via the “limma” R package. We set *P*-adjusted value < 0.05 and |log2 fold change (logFC)| >1 as the threshold of DEGs. The intersection of the GSE83456 positively correlated module genes with the upregulated differentially expressed genes of GSE42826, GSE42830, and GSE42834 was believed to be the key upregulated genes of sarcoidosis. Similarly, the intersection of the GSE83456 negatively related module genes with the downregulated differentially expressed genes of GSE42826, GSE42830, and GSE42834 was thought to be the key downregulated genes of sarcoidosis.

### LASSO machine learning algorithm

The least absolute shrinkage and selection operator (LASSO) was performed to obtain a robust diagnostic performance model. LASSO is a popular algorithm that is broadly utilized in medical studies (16–18). 10-fold cross-verification was performed to determine the Lambda minimum. This machine learning algorithmic procedure was implemented with the “glmnet” R package. In addition, LASSO can obtain relevant genes for the diagnosis of sarcoidosis for further mechanistic studies. The receiver operator characteristic (ROC) curves were generated, and the area under the ROC curve (AUC) assessed the performance of the disease diagnostic model.

### Gene set enrichment analysis

The normalized enrichment scores (NES) were calculated for sarcoidosis based on the diagnostic model scores on GO terms and KEGG pathway in the Molecular Signature Database (MSigDB) via all GO gene sets (c5.go.v7.4.symbols.gmt) and KEGG gene sets as Gene Symbols (c2.cp.kegg.v7.4.symbols.gmt), respectively. We set |NES| > 1.50, and *P*-adjusted value < 0.01 as cutoff criteria.

### Evaluation of immune landscape

Single sample GSEA (ssGSEA) (19) that generates enrichment scores for a single sample was used to explore differences in immune cell infiltration between sarcoidosis and normal samples. The abundance of the 24 immune infiltrating cells was calculated and visualized by the “GSVA” R package (v1.42.0). In addition, correlation coefficients between the diagnostic model scores and the immune cell abundance of the samples were calculated to investigate the significant immune cells involved in sarcoidosis and the immune mechanisms.

### Statistical analysis

Data processing, statistical analysis, and plotting were carried out in the R 4.1.2 software. The correlation between two continuous variables was assessed using Pearson's correlation coefficient. Comparisons of categorical variables were done using the Chi-square test, while comparisons of continuous variables were done using the Wilcoxon rank-sum test or *t*-test. *P*-value < 0.05 was determined to be statistically significant.

## Results

### Data acquisition from GEO

A total of 313 sarcoidosis patients from 11 public datasets were collected for further analysis (Supplementary Table 1). A total of 11 datasets were selected: six came from whole blood samples (GSE42834, GSE83456, GSE42826, GSE42830, GSE18781, and GSE34608), two came from PBMC samples (GSE19314 and GSE37912), two came from BAL cells samples (GSE73394 and GSE75023), and one came from lung tissue samples (GSE16538). The baseline characteristics can be found in Supplementary Table 2. The gene expression data of four datasets (GSE83456, GSE42834, GSE42826, and GSE42830) were used to screen the essential genes. The seven remaining datasets were used as the validation sets.

### Identification of key modules through WGCNA

**Figure 2:**
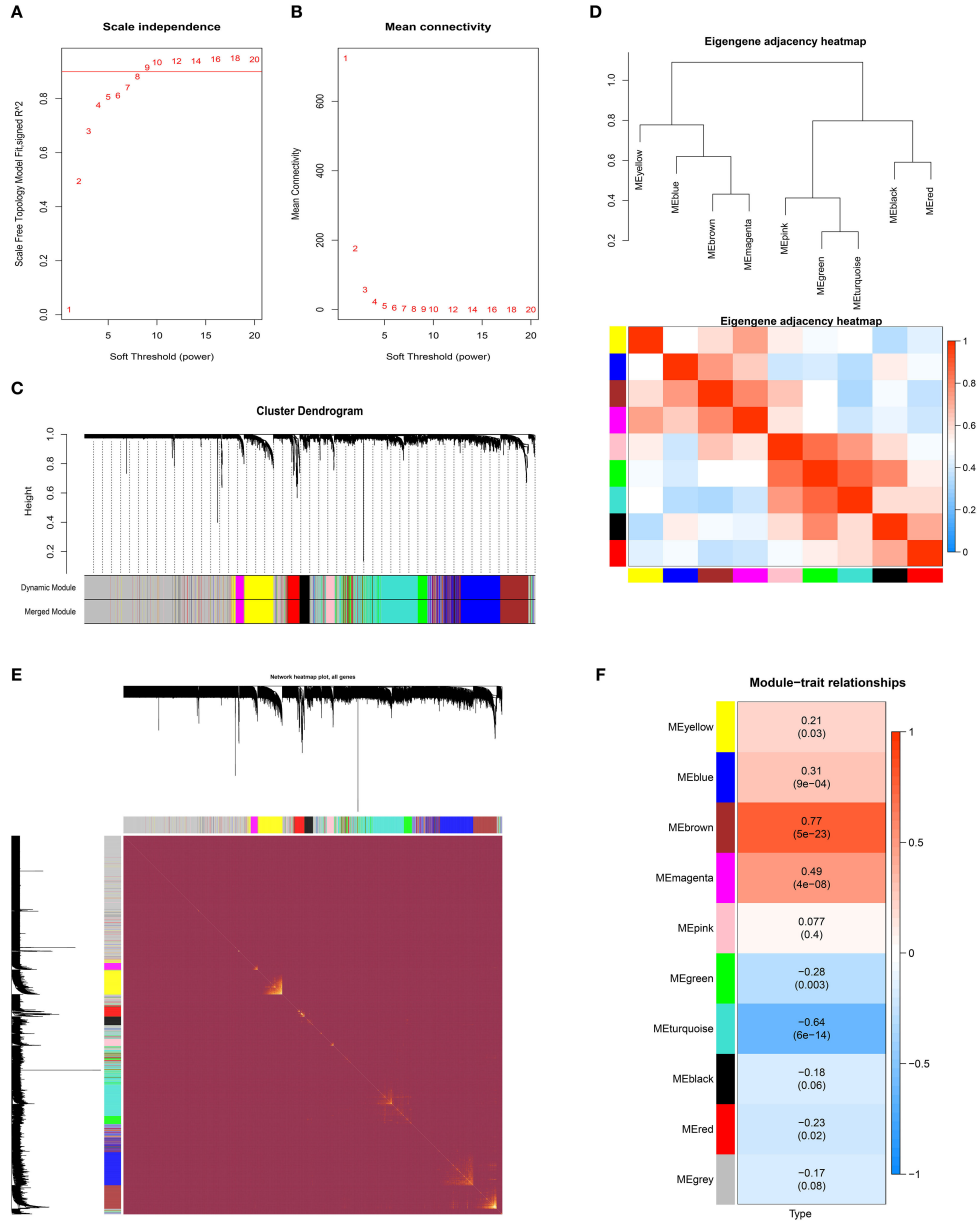
The weighted gene co-expression network analysis of sarcoidosis. **(A)** Scale-free topological model fit at various soft-thresholding powers. **(B)** Mean connectivity for different soft-thresholding powers of the network. **(C)** Gene clustering dendrograms based on hierarchical clustering under optimal soft-thresholding power. **(D)** The heatmap of the eigengene adjacency. **(E)** The heatmap of the topological overlap matrix of genes selected by WGCNA. **(F)** The relationships between MEs and sarcoidosis.

The GSE83456 dataset was used to identify the key MEs related to sarcoidosis. First, no outlier sample was removed based on the sample tree, and then totaling 110 samples, the top 5,000 genes were used for WGCNA. The soft-thresholding power was set to nine to fit the scale-free network (Figures 2A,B). Second, the gene similarity matrix was constructed as an adjacency matrix according to the Pearson correlation values. The adjacency matrix was converted to the TOM and 1-TOM, reflecting the similarity and dissimilarity between genes separately. Third, the co-expression modules in the network were identified using the “cutreeDynamic” function, and all genes were clustered into 10 modules. These modules were further merged to nine MEs using the “mergeCloseModules” function plotted by clustering dendrogram (Figure 2C) and heatmap of the eigengene adjacency (Figure 2D). Figure 2E shows the heatmap of the topological overlap matrix of genes selected by WGCNA. The relationships between the MEs and sarcoidosis were visualized in the module-trait relationship diagram (Figure 2F).

### Enrichment analyses of genes in key modules

**Figure 3:**
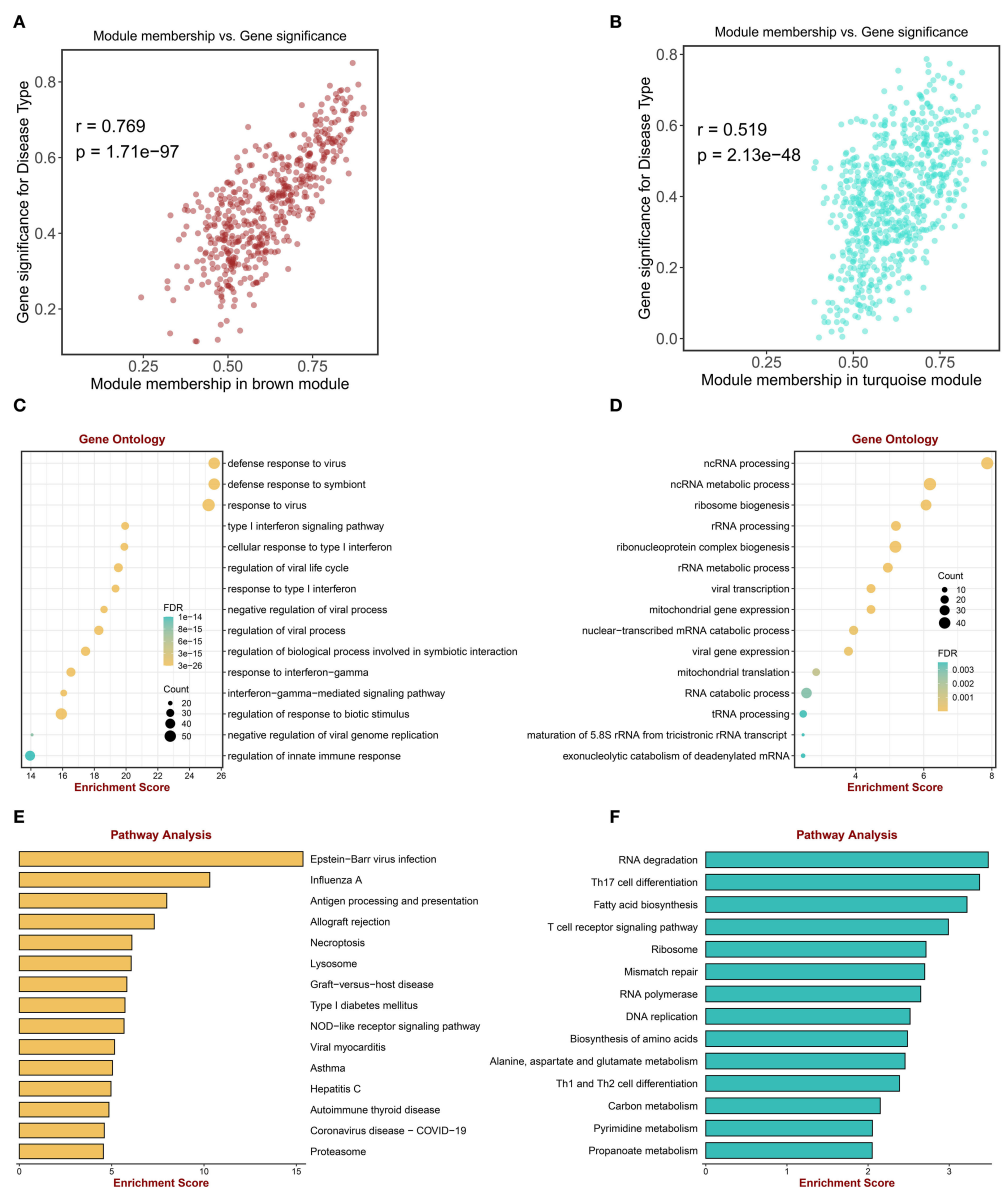
Enrichment analysis of genes in key MEs. **(A)** The scatter plot correlation between the brown module membership and gene significance. **(B)** The scatter plot of correlation between the turquoise module membership and the gene significance. **(C)** Go enrichment analysis of genes in the brown module. **(D)** GO enrichment analysis of genes in the turquoise module. **(E)** KEGG pathway analysis of genes in the brown module. **(F)** KEGG pathway analysis of genes in the turquoise module.

Among the nine modules, the brown module was the most positively correlated module with sarcoidosis, including 493 genes, and the correlation between module membership and gene significance was 0.769 (*P* < 0.0001) (Figure 3A). The turquoise module was the most negatively correlated module, including 684 genes and the correlation was 0.519 (*P* < 0.0001) (Figure 3B). Figure 3C displayed that the genes of the brown module were significantly enriched in “defense response to virus,” “defense response to symbiont,” “response to virus,” “type I interferon signaling pathway,” and “regulation of innate immune response” in GO terms. Figure 3D illustrated that the genes of the turquoise module were significantly enriched in “ncRNA processing,” “ncRNA metabolic process,” and “ribonucleoprotein complex biogenesis” in GO terms. The enriched KEGG pathways of the brown module genes, including “Epstein-Barr virus infection,” “Influenza A,” “Antigen processing and presentation,” and “Allograft rejection” were shown in Figure 3E. The enriched KEGG pathways of the turquoise module included the “RNA degradation,” “Th17 cell differentiation,” and “T cell receptor signaling pathway” (Figure 3F). The enrichment analysis results indicated that inflammatory and immune cells played an essential role in the process of sarcoidosis.

### Identification of DEGs

**Figure 4:**
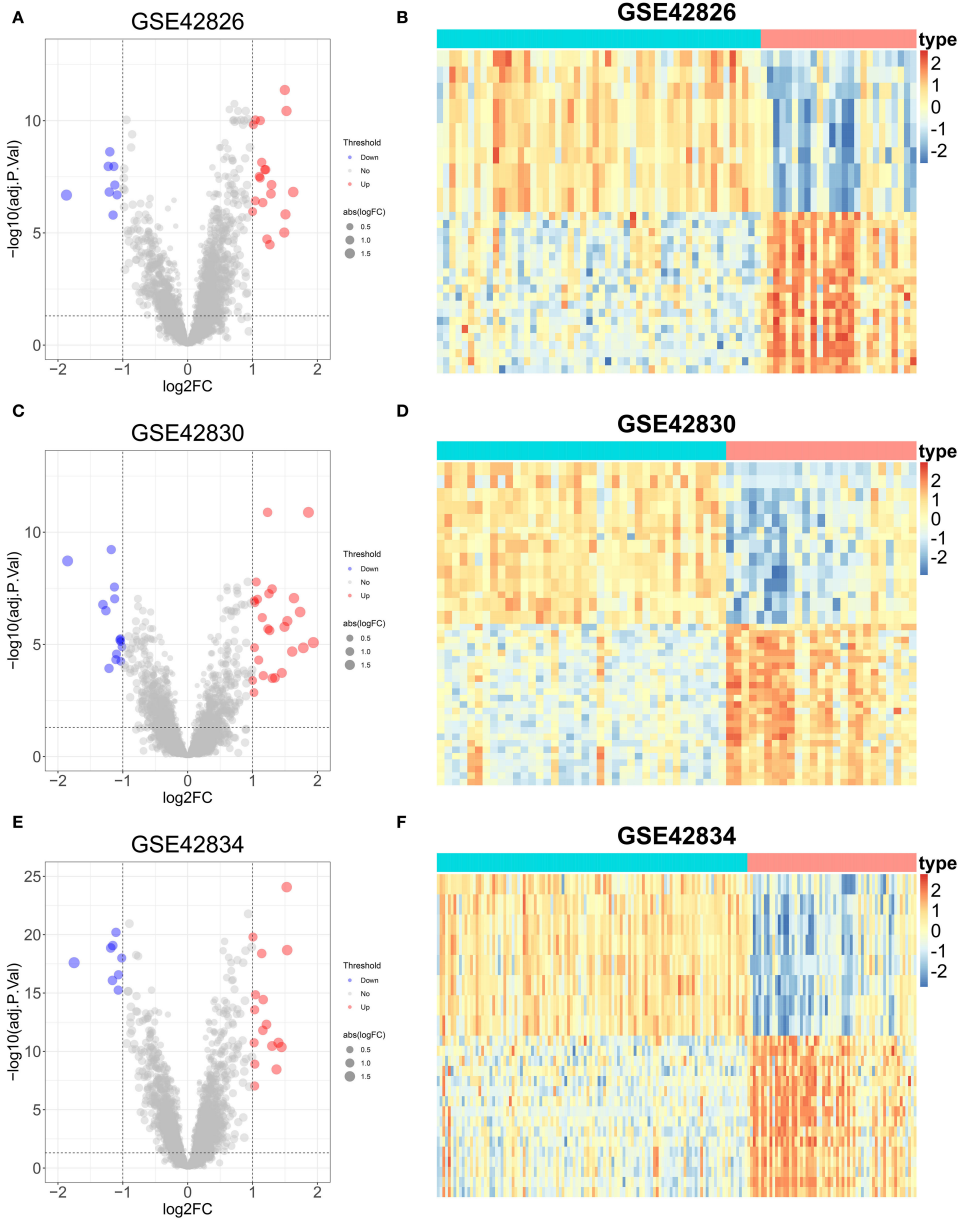
Differential expression genes analysis of three sarcoidosis datasets. **(A)** The volcano plot of DEGs in GSE42826. **(B)** The heatmap of the top 50 DEGs in GSE42826. **(C)** The volcano plot of DEGs in GSE42830. **(D)** The heatmap of the top 50 DEGs in GSE42830. **(E)** The volcano plot of DEGs in GSE42834. **(F)** The heatmap of the top 50 DEGs in GSE42834.

The DEGs between sarcoidosis and normal patients were explored by the “limma” R package. In the GSE42826 dataset, 20 significantly upregulated genes and eight significantly downregulated genes were defined, shown as a volcano plot and heatmap in Figures 4A,B. The GSE42830 dataset identified 26 considerably upregulated genes and 14 significantly down-regulated genes, shown in Figures 4C,D. Similarly, in the GSE42834 dataset, 16 upregulated genes and 8 significantly downregulated genes were defined, demonstrated as a volcano plot and heatmap in Figures 4E,F.

### Construction of a sarcoidosis diagnosis signature via machine learning

**Figure 5:**
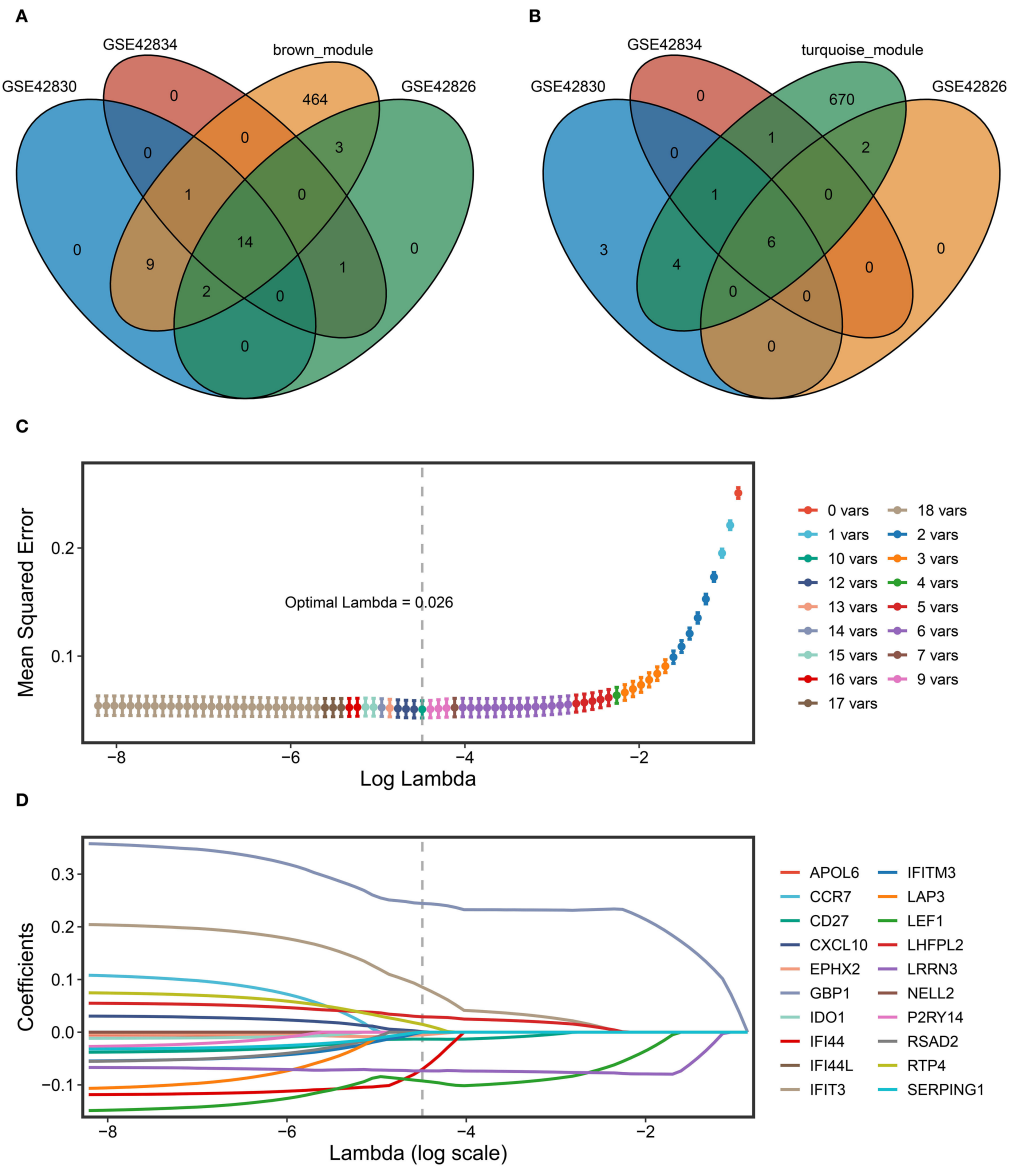
The construction of the SARDS based on the LASSO algorithm. **(A)** The Venn diagram of the intersection between the upregulated DEGs in three datasets and genes in the brown module. **(B)** The Venn diagram of the intersection between the downregulated DEGs in three datasets and genes in the turquoise module. **(C)** Determination of the optimal lambda was obtained when the partial likelihood deviance reached the minimum value, and further generated the key gene with nonzero coefficients. **(D)** LASSO coefficient profiles of the candidate gene for SARDS construction.

First, the key regulated genes were screened to further model construction by the intersection of the genes in the key module of WGCNA and the significantly regulated genes in the three datasets. Fourteen key upregulated genes were screened through the intersection of the brown module genes of WGCNA and the significantly upregulated genes of the three datasets (Figure 5A). Likewise, six key downregulated genes were screened through the intersection of the turquoise module genes and the significantly downregulated genes of three datasets (Figure 5B). A total of 20 key genes have been exploited as stable and reliable sarcoidosis diagnostic signatures (SARDS) to diagnose sarcoidosis at the gene level by applying the LASSO algorithm. The optimal lambda was 0.026 when the LASSO regression partial likelihood deviation was minimized (Figure 5C). Therefore, 10 key genes with non-zero LASSO coefficients were considered the main variables in the diagnostic model (Figure 5D). The 10 genes were *GBP1, LEF1, IFIT3, LRRN3, IFI44, LHFPL2, RTP4, CD27, EPHX2*, and *CXCL10* with the coefficients 0.244, −0.0925, 0.0855, −0.0732, −0.0703, 0.0292, 0.0149, −0.0131, −0.00522, and 0.000941, respectively. The SARDS was established with the following formula: SARDS score = 0.445 + 0.244 × Exp *GBP1*- 0.0925 × Exp *LEF1*- 0.0855 × Exp *IFIT3*- 0.0732 × Exp *LRRN3*- 0.0703 × Exp *IFI44* + 0.0292 × Exp *LHFPL2* + 0.0149 × Exp *RTP4*- 0.0131 × Exp *CD27*- 0.00522 × Exp *EPHX2*- 0.000941 × Exp *CXCL10*.

### SARDS validation in different cohorts

**Figure 6:**
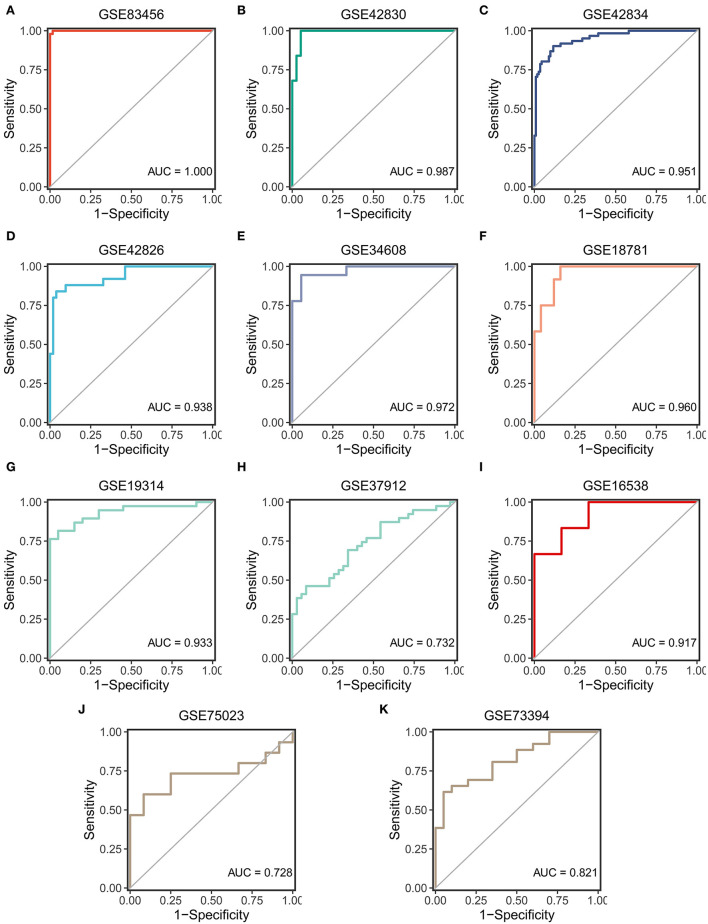
The validation of the SARDS in 11 cohorts. **(A)** The ROC curve of the modeling dataset (GSE83456). **(B–K)** The ROC curves of validation datasets (GSE42830, GSE42834, GSE42826, GSE34608, GSE18781, GSE19314, GSE37912, GSE16538, GSE75023, and GSE73394).

ROC curves were used to assess the diagnostic efficacy of SARDS in 11 cohorts. The GSE83456, as the training cohort, performed an excellent AUC of 1.00 (Figure 6A). The GSE42830, GSE42834, and GSE42826, which were involved in screening the key genes, performed outstanding AUCs of 0.987, 0.951, and 0.938 (Figures 6B–D). The GSE34608 and GSE18781, as the validation dataset of whole blood samples, had superior diagnostic efficacy in that AUCs were 0.972 and 0.960 (Figures 6E,F). Meanwhile, the cohorts with samples of PBMC, BAL cells, and lung tissue were enrolled in the validation cohort of the SARDS. The GSE19314 and GSE37912 of PBMC samples performed AUCs of 0.933 and 0.732 (Figures 6G,H). The GSE16538 of lung tissue had an AUC of 0.917, shown in Figure 6I. The GSE75023 and GSE73394 displayed the great AUCs of 0.728 and 0.821 (Figures 6J,K). The SARDS has been proven to be a robust and reliable diagnostic model of sarcoidosis.

### Exploration of mechanisms based on the SARDS score

**Figure 7:**
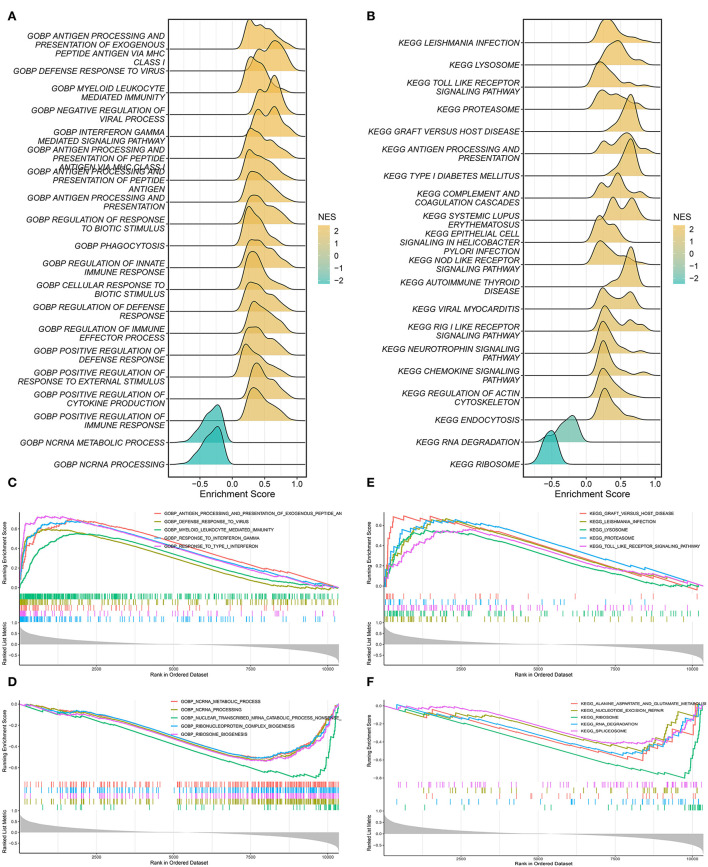
Gene set enrichment analysis. **(A)** The ridge plot of the top 20 GO terms with ranked genes of the GSE83456. **(B)** The ridge plot of the top 20 KEGG pathways with ranked genes of the GSE83456. **(C,D)** The positive and negative top five GO terms with ranked genes of the GSE83456. **(E,F)** The positive and negative top five KEGG pathways with ranked genes of the GSE83456.

The correlation between SARDS scores and gene expression was calculated for gene sequencing to detect latent mechanisms of sarcoidosis by applying GSEA. The most important GO terms and the KEGG pathways were displayed in the ridge plot (Figures 7A,B). Among these, Figure 7C depicted the top five positively relevant GO terms, including “Response to interferon gamma,” “Response to type I interferon,” “Antigen processing and presentation of exogenous peptide antigen via MHC class I,” “Defense response to virus,” and “Myeloid leukocyte mediated immunity.” Figure 7D depicted the top five negatively relevant GO terms, comprising “Nuclear transcribed mRNA catabolic process nonsense mediated decay,” “ncRNA processing,” “Ribosome biogenesis,” “Ribonucleoprotein complex biogenesis,” and “ncRNA metabolic process.” On the other hand, Figure 7E described the top five positively correlated KEGG pathways, consisting of “Leishmania infection,” “Lysosome,” “Toll like receptor signaling pathway,” “Proteasome,” and “Graft vs. host disease.” Likewise, Figure 7F described the top five negatively correlated KEGG pathways, consisting of “Ribosome,” “RNA degradation,” “Alanine aspartate and glutamate metabolism,” “Nucleotide excision repair,” and “Spliceosome.”

### The immune landscape of sarcoidosis

**Figure 8:**
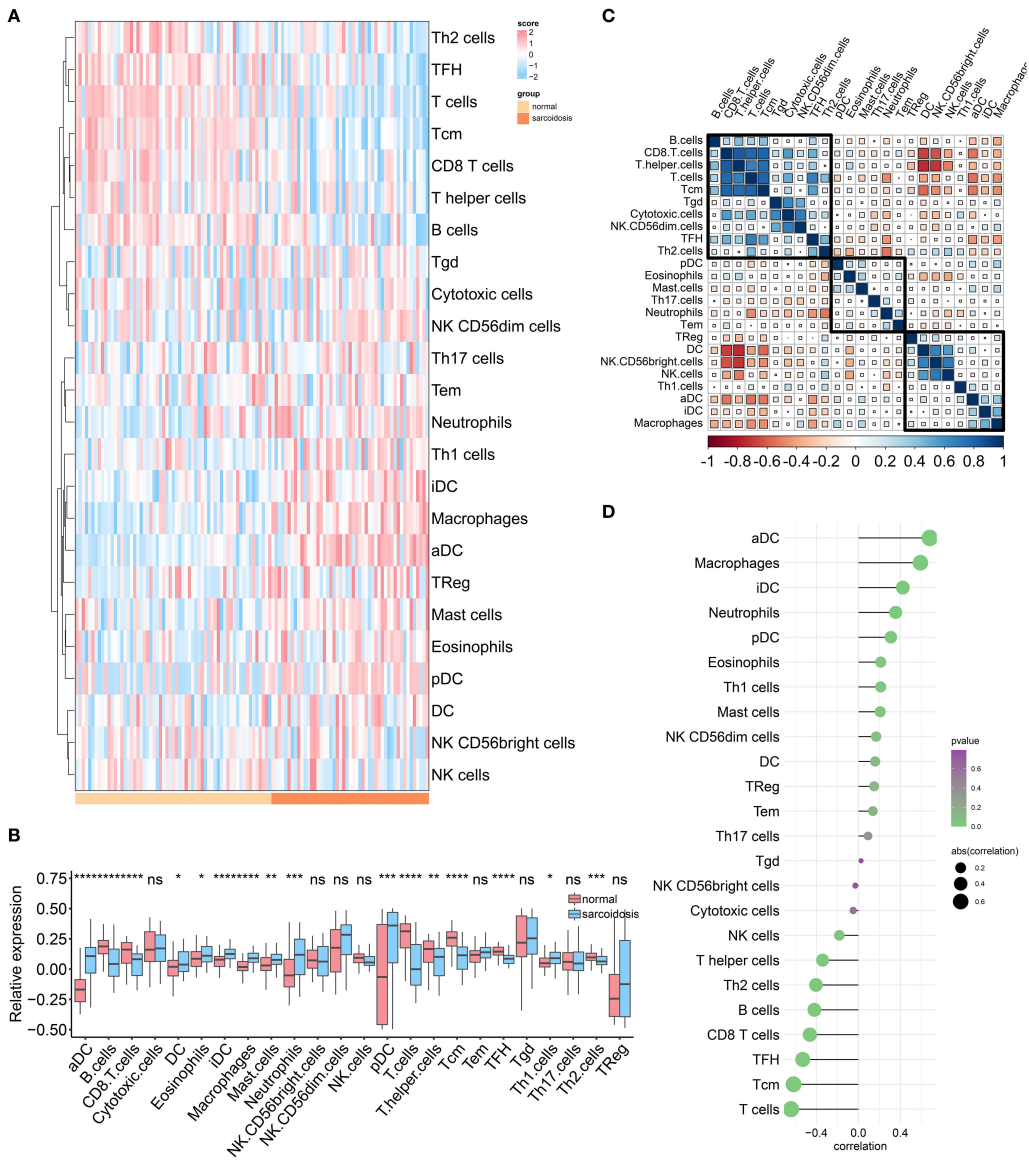
The immune landscape of sarcoidosis. **(A)** The heatmap of the immune infiltration in sarcoidosis and normal groups. **(B)** The boxplot of the 24 types of immune cell infiltration in sarcoidosis and normal groups. *P < 0.05, **P < 0.01, ***P < 0.001. **(C)** The heatmap of the correlations between different immune cells. **(D)** The relationship between the SARDS score and immune infiltration.

Given that sarcoidosis is a systemic inflammatory disease of unknown mechanisms, it is essential to exploit the immune microenvironment of sarcoidosis patients. The ssGSEA algorithm was performed to estimate the infiltration abundance of 24 types of immune cells between sarcoidosis and normal patients. The heatmap and boxplot demonstrated the fraction and expression differences of 24 types of immune cells in the GSE83456 cohort (Figures 8A,B). It was evident that the superior abundance of the anchorage-dependent cell (aDC), macrophages, immature dendritic cells (iDC), neutrophils, plasmacytoid dendritic cells (pDC), eosinophils, Th1 cells, and mast cells and the inferior infiltration of T cells, Central Memory T cell (Tcm), T follicular helper cell (TFH), CD8 T cells, B cells, Th2 cells, and T helper cells were the immune signatures of the sarcoidosis patients. The correlations between different immune cells were shown in the heatmap (Figure 8C). The T helper cells and CD8 T cells showed the strongest positive correlation, and DC and T helper cells showed the strongest negative correlation. The correlation between the SARDS score and immune infiltration was shown in Figure 8D. We can see that the infiltration level of aDC cells (*r* = 0.680, *P* < 0.0001), macrophages (*r* = 0.591, *P* < 0.0001), iDC (*r* = 0.423, *P* < 0.0001), neutrophils (*r* = 0.355, *P* = 0.0001), pDC (*r* = 0.309, *P* = 0.0011), and eosinophils (r = 0.213, *P* = 0.0260) were positively correlated with the SARDS score; the infiltration level of T cells (*r* = −0.643, *P* < 0.0001), Tcm (*r* = −0.618, *P* < 0.0001), TFH (*r* = −0.531, *P* < 0.0001), CD8 T cells (*r* = −0.463, *P* < 0.0001), B cells (*r* = −0.420, *P* < 0.0001), and Th2 cells (*r* = −0.405, *P* < 0.0001) were negatively associated with the SARDS score.

## Discussion

The diversity of clinical symptoms in sarcoidosis and the lack of a single reliable diagnostic criterion make prompt and accurate diagnosis challenging. In addition, the etiology and pathogenesis of sarcoidosis remain unknown, making treatment available considerably limited. Therefore, exploring the latent mechanisms and constructing an accessible and reliable diagnostic model of sarcoidosis is vital for innovative therapeutic approaches to improve prognosis.

Our study was based on gene transcriptome analysis of 303 sarcoidosis samples and 400 normal controls. Two modules with the highest correlation to sarcoidosis were identified through WGCNA, namely brown and turquoise modules. The brown module, containing 493 genes, demonstrated that the defense response to the virus and innate immune response might play an essential role in the pathogenesis of sarcoidosis, which was consistent with the results of previous studies. Numerous immunological arguments had indicated that inadequate clearance of viral particles, accompanied by various immunodeficiencies, might be relevant to sarcoidosis disease (20). In patients with sarcoidosis, viruses have evolved strategies to evade or suppress host cell defenses utilizing the process of autophagy (20). The primary regulator of the innate immune response in sarcoidosis was the alveolar macrophage, which both produced pro-inflammatory cytokines, such as tumor necrosis factor-α (TNF-α), contributing to the production of granulomatous lesions, and acted as an antigen-presenting cell (APC) interacting with T cells via human leucocyte antigen (HLA) molecules and T cell receptors (21, 22). The turquoise module, containing 684 genes, revealed that non-coding RNA (ncRNA), including processing, metabolic processes and degradation, and immune cells, was strongly associated with sarcoidosis through enrichment analysis. Regulative ncRNAs can be classified into microRNAs (miRNAs), long ncRNAs (lncRNAs), and small interfering RNAs. Previous research has indicated potential associations between the dysregulation of some miRNAs and the diagnosis and prognosis of sarcoidosis (23, 24). In PBMCs from patients with sarcoidosis, levels of miRNA-34a were increased, which downregulated sirtuin (SIRT) 1 and stimulated the secretion of INF-γ (25). SIRT1 is an essential mediator of energy metabolism and tissue survival, and INF-γ is necessary to develop and maintain the sarcoidosis status. These all contributed to the NF-κB-mediated inflammatory response in patients with sarcoidosis (26). In addition, miR-let7f, miR-15b, miR-16, miR-20a, miR-27b, miR-128a, miR-130a, miR-192, miR-221, and miR-222, miRNAs that target genes involved in angiogenesis and extracellular matrix remodeling, were found differentially expressed in a study between patients with sarcoidosis and controls (27). These genes were essential in the pathogenesis of sarcoidosis, including granuloma formation and fibrosis. Further research into the function of ncRNAs and immune cells in disease enhanced the understanding of the pathogenesis of sarcoidosis and provided new perspectives for translation into innovative therapeutic strategies (28).

Multiple reliable bioinformatics approaches were performed in our study to screen for essential molecular biomarkers associated with sarcoidosis. The intersection of WGCNA-associated module genes and differentially expressed genes was considered the critical gene, most strongly correlated with sarcoidosis and significantly differentially expressed in other sarcoidosis cohorts. A total of 20 genes were screened for dimensional reduction to construct the diagnostic model, and 10 genes were ultimately identified to the SARDS using the LASSO algorithm, including *GBP1, LEF1, IFIT3, LRRN3, IFI44, LHFPL2, RTP4, CD27, EPHX2*, and *CXCL10*. *GBP1*, as an IFN-γ-related gene in whole blood gene expression, was independently and positively correlated with T-bet+ frequency in Th17 cells, as the expression of T-bet in Th17.0 cells might indicate the degree of granulomatous inflammation in sarcoidosis patients (29). This result was consistent with previous enrichment analyses, demonstrating that IFN-γ and Th17 cells had an essential effect on the development and progression of granulomatous tissues in sarcoidosis (30, 31). Lymphoid enhancer-binding factor 1 (*LEF1*) is one of the Hippo signaling pathway hub genes, and it has been suggested that macrophage proliferation is related to the downregulation of the Hippo signaling pathway (32). The pathology of sarcoidosis is characterized by chronic granulomas with a core infiltration of macrophages and a peripheral infiltration of lymphocytes visually (33). Therefore, the downregulation of *LEF1* contributed to the diagnosis of sarcoidosis in our study. The single-cell analysis identified a new sub-group of macrophages called IFN-responsive macrophages (IFNRM) that expressed IFN-responsive genes (such as *IFIT3*) and secreted the cytokine CXC motif chemokine 10 (*CXCL10*), which regulated the proliferation and differentiation of satellite cells (34). On the question of *CD27*, the research found that the abundant B-cell infiltration in granuloma tissue indicated that B cells were directly involved in the inflammatory process in patients with sarcoidosis. And *CD27*(-) B cells may be a biomarker for treatment with TNF-α blocking agents. In addition, we found that *LRRN3, IFI44, LHFPL2, RTP4*, and *EPHX2* were all involved in diagnosing sarcoidosis, which might shed light on the mechanisms of sarcoidosis and provide potential biomarkers for diagnosis. Overall, results from 11 different cohorts of whole blood, PBMC, BAL cells, and lung tissue supported the diagnostic efficacy of the essential genes, with the splendid AUCs ranging from 0.938 to 1.000 in training datasets and ranging from 0.728 to 0.972 in validation datasets. He J et al. found that *BATF2* and *PDK4* could be used as diagnostic molecular markers for sarcoidosis through bioinformatics approaches in two cohorts (35). Our SARDS model, which combined the construction and validation of 11 cohorts, had higher diagnostic efficacy than other diagnostic models, further validating that SARDS was feasible and reliable in diagnosing patients with sarcoidosis.

Considering that both functional enrichment analysis and GSEA results based on SARDS scores indicated the involvement of immune cells and their processes in sarcoidosis, it was essential to explore the immune landscape in patients with sarcoidosis. This study found that the high infiltration of iDCs, macrophages, pDCs, neutrophils, and eosinophils and the low infiltration of T cells, Tcm, TFH, CD8 T cells, B cells, and Th2 cells constituted the immune microenvironment of sarcoidosis. As we know, dendritic cells comprise three lineages including myeloid DC (mDC), pDC, and Langerhans cells (LC). In the immune process of sarcoidosis, dendritic cells migrate to lymph nodes and participate in t-cell proliferation through t-cell receptors and costimulatory molecules (36). Subsequently, alveolar macrophages are activated to secrete TNF chemotactic leukocytes, which promote granuloma formation (21). The iDCs were enriched in BALF and skin lesions of patients, while mature DCs were located in lymph nodes (37). The pDCs resemble lymphocytes that produce large amounts of interferon-alpha (IFN-α) upon viral invasion, which is consistent with the results of the enrichment analysis regarding the viral response. Taken together, these results suggest that TNF is an important mediator in the pathogenesis of sarcoidosis. More surprisingly, TNF receptors are also abundant in DCs, making DCs possible for therapeutic targets. Following the present results, previous studies have demonstrated that patients with sarcoidosis had strong immuno-stimulability of DCs and macrophages in both the lung and blood (38, 39).

Overall, our research had limitations and strengths. The limitations of the study were that the cohorts in our study contained only diagnostic information lacking clinical aspects. Besides, the essential genes screened were not validated experimentally. Further studies need to be carried out to validate the value of the clinical application. However, it had the strength of a sufficiently large sample size of sarcoidosis, containing 313 patients and 400 healthy controls in 11 cohorts with four different sample sources. Validation in various sample sources and diverse cohorts compensated for experimental validation. In addition, advanced bioinformatics methods and machine learning algorithms reduce the impact of disease heterogeneity and confounding factors on diagnostic models. Collecting and analyzing circulating cells, indicative of pathogenic mechanisms and immune characteristics, are less invasive and less costly for new diagnostic tools.

In summary, our study systematically identified a feasible and credible diagnostic signature (termed SARDS) comprising 10 essential molecular biomarkers for the diagnosis of sarcoidosis and validated its robustness and translation in multiple cohorts of different source types. The study also had significant implications in exploring the underlying pathogenesis and the immune landscape of sarcoidosis for innovative therapeutic strategies. Taken together, SARDS could be a promising tool to optimize the diagnosis and treatment of patients with sarcoidosis.

## Data availability statement

The datasets presented in this study can be found in online repositories. The names of the repository/repositories and accession number(s) can be found in the article/Supplementary material.

## Author contributions

MD, ZL, and ZC designed the research. MD and ZL performed data acquisition and data analysis. PL, YW, and YuZ assisted with data analysis. MD wrote this manuscript. SW, YoZ, MF, RW, HX, and YR edited and revised this manuscript. All authors read and approved the manuscript.

## Funding

This study was supported by the National Natural Science Foundation of China (U1904142 and 82170037) and the Medical Science and Technology Research Project of Henan Province (SBGJ202002043).

## Conflict of interest

The authors declare that the research was conducted in the absence of any commercial or financial relationships that could be construed as a potential conflict of interest.

## Publisher's note

All claims expressed in this article are solely those of the authors and do not necessarily represent those of their affiliated organizations, or those of the publisher, the editors and the reviewers. Any product that may be evaluated in this article, or claim that may be made by its manufacturer, is not guaranteed or endorsed by the publisher.
